# Adherence of Internet-Based Cancer Risk Assessment Tools to Best Practices in Risk Communication: Content Analysis

**Published:** 2021-01-25

**Authors:** Erika A Waters, Jeremy L Foust, Laura D Scherer, Amy McQueen, Jennifer M Taber

**Affiliations:** 1 Washington University School of Medicine Saint Louis, MO United States; 2 Kent State University Kent, OH United States; 3 University of Colorado School of Medicine Denver, CO United States

**Keywords:** health communication, personalized medicine, internet, risk assessment

## Abstract

**Background:**

Internet-based risk assessment tools offer a potential avenue for people to learn about their cancer risk and adopt risk-reducing behaviors. However, little is known about whether internet-based risk assessment tools adhere to scientific evidence for what constitutes *good* risk communication strategies. Furthermore, their quality may vary from a user experience perspective.

**Objective:**

This study aims to understand the extent to which current best practices in risk communication have been applied to internet-based cancer risk assessment tools.

**Methods:**

We conducted a search on August 6, 2019, to identify websites that provided personalized assessments of cancer risk or the likelihood of developing cancer. Each website (N=39) was coded according to standardized criteria and focused on 3 categories: general website characteristics, accessibility and credibility, and risk communication formats and strategies.

**Results:**

Some best practices in risk communication were more frequently adhered to by websites. First, we found that undefined medical terminology was widespread, impeding comprehension for those with limited health literacy. For example, 90% (35/39) of websites included technical language that the general public may find difficult to understand, yet only 23% (9/39) indicated that medical professionals were their intended audience. Second, websites lacked sufficient information for users to determine the credibility of the risk assessment, making it difficult to judge the scientific validity of their risk. For instance, only 59% (23/39) of websites referenced the scientific model used to calculate the user’s cancer risk. Third, practices known to foster unbiased risk comprehension, such as adding qualitative labels to quantitative numbers, were used by only 15% (6/39) of websites.

**Conclusions:**

Limitations in risk communication strategies used by internet-based cancer risk assessment tools were common. By observing best practices, these tools could limit confusion and cultivate understanding to help people make informed decisions and motivate people to engage in risk-reducing behaviors.

## Introduction

### Background

Epidemiological risk prediction models use a patient’s medical history, demographic characteristics, and/or behaviors to estimate their likelihood of experiencing a variety of clinical outcomes, such as disease incidence, progression, and survival [1]. The basic premise of many of these models is that providing people with personalized risk information will foster informed decision making (eg, engage in risk-stratified cancer screening [2,3] and take medications to prevent cancer [4]) and encourage healthy lifestyle behaviors (eg, increase physical activity engagement [5]). Furthermore, such risk models are central to a key precision medicine goal of making medical decisions based on personalized risk data [6,7]. Before the internet became widely accessible by the general public, the use of these models was restricted to clinical and research settings [4,8]. With the advancement of computing and internet technology, it has become easier for researchers to translate risk prediction models into tools that allowed for the rapid assessment of an individual’s risk (hereafter, *risk assessment tool*). In 2009, a content analysis reported finding 47 websites that contained cancer-specific risk assessment tools by simply entering keywords into common search engines [9]. Since then, the number of cancer risk prediction models that could potentially be translated into risk assessment tools has dramatically increased. From 1993 to 2009, an average of 4 models were published and indexed on PubMed each year; this number increased to an average of 40 per year from January 2010 to August 2020 [10]. However, despite the increased number of risk prediction models, little is known about how internet-based risk assessment tools have (or have not) changed in the last decade.

### Public-Facing Risk Assessment Tools Need to Use Good Communication Practices

The US National Cancer Institute (NCI) created the Health Information National Trends Survey (HINTS) in response to a paucity of data about the US population’s cancer information needs and cancer information–seeking experiences [11]. The NCI launched the first survey in 2003, and additional iterations were administered approximately every 2 years. Data from HINTS have revealed that, from 2003 to 2019, the percentage of US residents who reported having ever sought cancer information rose from 45% to 54% [12]. More strikingly, those who used the internet as their first source of cancer information rose from approximately 46% to 75% [13,14]. Unfortunately, not only have many information seekers had negative experiences during their search, but such experiences have not improved from 2003 to 2018. Approximately 60% to 65% of HINTS respondents reported that the information was difficult to understand [15] or they were frustrated during the search process [14]. Over half of them reported concerns about the quality of the information [16].

The 2009 content analysis of internet-based cancer risk assessment tools also identified serious concerns about the quality of experiences that people might have had while searching for personalized risk assessment tools [9]. One major limitation was related to the accessibility of the information to the general public. Specifically, using medical terminology like *biopsy* without explaining what the terms meant likely limited accessibility to individuals with limited health literacy. Similarly, few tools provided an option for people who spoke a language other than English to obtain information in their native language.

The second major limitation was that only half of the websites provided information needed to evaluate their credibility, such as a citation of a peer-reviewed journal or a description of the risk prediction model used to calculate personalized estimates [17,18]. Without this information, it would be reasonable for website users to doubt the credibility of the information.

The third major category of limitations was how the actual risk information was communicated. Although most websites in the 2009 review adhered to some general principles, such as communicating numerical information as percentages or frequencies (ie, 6 in 100) and providing advice for reducing risk (eg, stop smoking), the use of other important risk communication principles was limited. Fewer than half of websites supplemented numeric estimates with qualitative labels (eg, *very high*), provided a visual display, or indicated the timeframe for which the risk was relevant (eg, 5-year risk of breast cancer) [19] (Table 1).

**Table 1:** Recommended risk communication formats.

Risk communication format and selected relevant citations	Why the format is important
1. Specify the duration of risk [20,21]	Time is an integral element of many risk prediction algorithms. Estimates for 1-year, 5-year, and lifetime risks can differ greatly. Consequently, specifying whether the risk estimate is applicable to the next 1 year, 5 years, 10 years, or lifetime is critical for helping people contextualize the risk and determine how much they should be concerned about it in the immediate future. For example, a 7% risk of breast cancer would be more worrisome if it was applicable to the next 5 years (in which case, it would be considered a high risk) than over one’s lifetime (in that case, it would be considered a low risk). [22]
2. Provide absolute risk estimates [23,24]	Absolute risk represents the likelihood that an individual will develop a disease. It is the most basic element of risk information people need to understand their risk. Absolute risk can be communicated as numbers (eg, 12%) or as qualitative labels (eg, “very high”).
3. When communicating numeric risk estimates, use percentages or simple frequencies (eg, N in 100)^a^. Do not include the 1 in N format, NNT^b^, or odds ratios [25-28]	Risk comprehension is highest when risk estimates are presented as a percentage or as simple frequencies (eg, N in 100). However, both recommended formats have drawbacks. The N in 100 format can encourage people to overemphasize risk by “imagining the numerator,” but the percentage format is more difficult to use while conducting complex calculations (eg, the probability of a woman having breast cancer given a positive mammogram). The 1 in N and NNT formats are very difficult to use and should be avoided.
4. When communicating numeric risk estimates, also include qualitative categories (also referred to as verbal labels; eg, high risk) [19,20,25,28,29]	Providing only numbers is problematic because of the population’s low levels of numeracy (ie, the ability to use numeric information) and a lack of contextual information (eg, should a 7% lifetime risk of breast cancer be considered a high risk or a low risk?). Providing qualitative categories (also, “verbal labels”) yields contextual information needed to interpret the importance of the numeric information. However, qualitative categories should be used in the absence of numeric information only with caution; people interpret qualitative categories as representing a wide range of possible probabilities (eg, “small” can mean “2%” to some people and “10%” to others). Qualitative categories can also prompt people to overestimate their risk. Providing both numeric estimates and qualitative categories yields both the detailed and contextual knowledge needed for informed decisions.
5. Frame the risk in positive and negative terms [20,21,25,30]	Framing the risk in negative terms only (eg, “Your risk of cancer is 5%”) places focus only on the negative outcome and might result in exaggerated risk perceptions. Adding positive framing (eg, “This means you have a 95% chance of not getting cancer”) helps participants place the risk in context.
6. If relative risk estimates are provided, then supplement it with absolute risk estimates [20,25,31]	Providing relative risk information in the absence of absolute risk information could lead people to believe that they will receive more benefit (or harm) from the action or treatment than what is possible (eg, a 50% risk reduction is much less impactful when the individual is at 4% absolute risk than at 40% absolute risk).
7. Include a visual display that depicts the individual’s risk [19,20,25,28,32]	Using a visual display can increase the comprehension of risk information. However, care must be taken to avoid biasing perceptions of risk (eg, displays that focus attention on the number of people affected by a disease can exaggerate a risk as compared with displays that include information about the number of people affected and the number of people who are not affected). In addition, some visual displays are more difficult for people to understand than others (eg, pie charts).
8. Acknowledge that the risk estimate contains an element of uncertainty [25,28]	Personalized risk estimates are based on the statistical modeling of population-level data. Consequently, they always contain a level of uncertainty due to (a) limitations in the reliability or adequacy of the information used to specify the risk prediction model or (b) the randomness of future events. Informing the audience that the risk estimate is just that—an estimate—is essential to prevent them from attributing an unreasonable degree of certainty to the estimate.
9. Provide information about risk reduction strategies [23,33,34]	Understanding how to reduce risk is an essential component of understanding one’s risk. Informing people how to reduce their risk is an important component of risk communication messages, particularly for individuals who have not learned risk reduction strategies previously. Providing risk information without such safety messages may undermine risk communication efforts by encouraging people to control their fear (eg, by trying to ignore the risk) rather than encouraging people to control the danger (eg, by engaging in appropriate health behaviors).
10. Consider providing comparative risk information (also called “social comparison” information) [23,28,35] but see a different perspective in studies by Fagerlin et al and Janssen et al [36,37]	Helping people understand how their risk compares to the risk of the average person of their age and sex may help them place their risk in context and evaluate its meaning and importance. For example, telling a woman that she has a 5% 5-year risk of developing breast cancer might not be meaningful unless she recognizes that it means that she is at above average risk. However, this can backfire; telling people only that they are at below-average risk might reduce motivation to engage in preventive behavior. [36]
11. Consider comparing the estimated risk to the risk of other hazards [23,28], but see different perspective in studies by Fischhoff and Covello [38,39]	Helping people understand where their risk of cancer falls in relation to other hazards such as heart disease, diabetes, or being in a car accident allows them to place the risk in context and thereby helps them determine where to invest their limited time, energy, and economic resources. However, care should be taken to avoid being perceived as condescending [38,39], lest the risk information be rejected.

^a^One controversial strategy sometimes used by risk assessment tools that aim to encourage healthy lifestyle behaviors is to communicate absolute risk information by only using qualitative (also referred to as verbal) labels [37,40] and to exclude numerical estimates [37,40]. This idea originates from the fact that numerical estimates that public health scientists would consider high may be perceived as quite low by the public, even when a verbal label is added. For example, a person who has a 3% risk of developing lung cancer in the next 6 years would be considered high risk [41]. However, research suggests that people may consider anything below 20% risk to be *low risk*, and anything between 20% and 70% to be *medium* risk [29]. Thus, it seems likely that a person advised to quit smoking because he or she has a 3% risk of lung cancer may decide that the effort involved in quitting is not *worth* the reduction of a very small risk. However, as noted in recommendation number 4, interpretations of qualitative labels are quite variable [42] and may result in an overestimation of risk [43]. In addition, providing quantitative information may increase trust among the general public [44].

^b^NNT: number needed to treat.

Since 2009, the scientific understanding of the complexities of risk communication—including what should be considered *good* risk communication strategies—has grown. Some principles remain the same, such as avoiding the 1 in X format, acknowledging uncertainty, including visual displays, using qualitative labels to provide context for numerical risk information, and providing advice for reducing risk [19,23,25,27,45,46]. However, one piece of new knowledge (or, rather, existing knowledge made explicit) is the importance of providing risk information in a way that is likely to achieve what the communicators wish to accomplish [47,48].

One goal of providing personalized risk information is to communicate information needed to make an informed decision about preference-sensitive treatment or screening options. Examples of such decisions include mammography screening at the age of 40-49 years, prophylactic mastectomy, chemopreventive medications to reduce breast cancer risk, prostate-specific antigen screening, lung cancer screening, and colorectal cancer screening modality (eg, fecal-occult blood test, fecal immunochemical test, colonoscopy, or sigmoidoscopy). These decisions necessitate communication strategies that allow users to evaluate information in a way that, to the greatest extent possible, fosters unbiased interpretations of the risks and benefits of each option [25,48,49].

Another goal of providing personalized risk information is to motivate people to engage in risk-reducing behaviors [5,48]. Such behaviors are not preference sensitive and, barring medical contraindications, are generally recommended for entire populations. For example, many public health agencies advocate reducing cancer risk by making universal, population-wide recommendations to avoid smoking tobacco; maintain a healthy weight; be physically active; limit alcohol intake; and engage in sun protection behaviors [50]. From this perspective, providing highly detailed risk and benefit information is not as important as conveying information in a way that alerts people to their general level of risk [48] and that convinces them to engage in risk-reducing actions [47]. However, it is also critically important to provide information in such a way that is simultaneously true and persuasive. Table 1 shows the risk communication formats and strategies that can be used for both informed decision making and persuasive contexts (items 1-9) and also offers suggestions for solely persuasive purposes (items 10-11).

### Study Objectives

From 2010 to 2020, many peer-reviewed articles and book chapters that describe current best practices in risk communication have been published [19,25,46,51]. In this study, we aim to understand the extent to which these practices have been applied to internet-based cancer risk assessment tools. To achieve this objective, we aim to (1) describe what risk communication formats and strategies internet-based cancer risk assessment tools use to facilitate their accessibility to the general public and demonstrate the credibility of their information, and (2) describe which risk communication formats and strategies internet-based cancer risk assessment tools use to present personalized risk information to the public.

## Methods

### Search Strategy and Website Selection

On August 6, 2019, we conducted an internet search to identify websites that provided people with personalized assessments of their cancer risk or the likelihood of developing cancer. One research assistant used Google and Bing (Microsoft) to search for the terms *calculate cancer risk*, *cancer risk calculator*, *estimate cancer risk*, *assess cancer risk*, and *cancer risk assessment*. Google and Bing accounted for over 80% of the world market share as of April 2020 [52]. She copied the URLs for the first 100 results for each of the 5 search terms for each of the search engines, which equaled 1000 results, into a Microsoft Excel spreadsheet. JF conducted an initial examination of these results and excluded 831 websites that were obviously not relevant (eg, risk of radiation from nuclear fallout). JF then more closely examined the remaining 169 results and excluded websites that were duplicates, redirected to another website already on the list (eg, a blog post that linked to a website already on the list), required a software download or installation of an app, had a link that no longer worked, did not provide personalized estimates of the risk of developing cancer, and/or required the user to provide medical information that must be obtained through consultation with a doctor (eg, prostate-specific antigen levels). When JF needed clarification about the appropriateness of a website, he consulted with EW; for these 13 websites, EW and JF discussed the elements of the website that were confusing and came to a decision by consensus. This process resulted in 37 websites being eligible for inclusion. Finally, we compared this list of 37 websites with the 47 websites included in the previous content analysis by Waters et al [9]. We found that 41 of the 47 websites from the 2009 analysis were no longer available. Only 6 active websites included in this review were also included in the 2009 review; 4 were identified during the search for this paper and 2 were located only by searching the 2009 paper. A total of 39 websites were included in this analysis (see Multimedia Appendix 1 for website URLs).

### Measures and Coding Procedures

A total of 3 broad categories of codes were examined: general website characteristics, accessibility and credibility, and risk communication formats and strategies. The coding categories, including descriptions and/or examples of each code, were developed based on the study by Waters et al [9]. For website characteristics, we coded cancer type as assessed by the tool and the type of organization that either developed or hosted the risk assessment tool. For accessibility and credibility, we coded aspects related to accessibility to the general public, aspects of website transparency, the apparent purpose of the website (ie, whether the website appeared to be intended to provide information, recruit users, or sell products), and whether and what type of details about where to seek more information were provided. For risk communication formats and strategies, we coded whether the duration of risk was specified, whether absolute and/or comparative information was provided, whether risk was communicated qualitatively or quantitatively, the format of quantitative information (eg, percent, N in 100) if relevant, whether positive framing was used, the type of visual display used if relevant, whether or how uncertainty was acknowledged, and whether the website included information about risk reduction strategies.

Each website was independently coded by 2 of the authors (EW and JF) according to the standardized coding criteria. After independently coding the first 5 websites, the coders discussed the results and revised the coding criteria as needed. The first 5 websites were independently coded again using the updated coding scheme, and the results were discussed. Then, the remaining 34 websites were independently coded by both the coders. Finally, the codes were reviewed by both the coders to identify any discrepancies. When discrepancies occurred, the coders discussed them until a consensus was reached. In the rare case where consensus could not be reached, the coders consulted another author (JT) to make a decision. As inter-coder agreement was obtained through inter-coder checks and consensus agreement, we do not report a quantitative calculation of kappa [53].

### Data Analysis

Simple frequencies were calculated and recorded for all the coding categories. We did not conduct significance testing because of the small sample sizes.

## Results

### General Website Characteristics

General website characteristics are summarized in Table 2. Altogether, the 39 websites provided users with up to 68 cancer risk estimates. Four websites allowed users to determine their risk of developing more than one type of cancer. In total, 16 cancer types were addressed by the calculators, with colon or colorectal, breast, and lung being the most common. The most common developer was the health care industry (eg, nonacademic-affiliated hospitals and insurance companies), which hosted just over one quarter of websites.

**Table 2:** General characteristics of internet-based cancer risk assessment tools (N=39 websites).

Website characteristic and description or examples of code^a^		Value, n (%)
**Cancer type^b^: the type of cancer for which the tool provided personalized risk estimates**		
	Colon or colorectal	13 (33)
	Breast	13 (33)
	Lung	10 (26)
	Melanoma	6 (15)
	Prostate	4 (10)
	Kidney	3 (8)
	Ovarian	3 (8)
	Pancreatic	3 (8)
	Cervical	2 (5)
	Stomach	2 (5)
	Uterine	2 (5)
	Cancer (general)	2 (5)
	Other^c^	5 (13)
**Website affiliation^d^: the type of organization that developed and/or hosted the tool**		
	Health care industry: nonacademic-affiliated hospitals or insurance companies (eg, Cigna, Merck, or Mayo Clinic)	10 (26)
	Government agency: any national or international governmental agency (eg, US National Cancer Institute)	6 (15)
	Educational institution: academic organization whose primary purpose is education (eg, University of California at Los Angeles)	6 (15)
	Independent medical expert: risk assessment tool was developed by a specific person who purports to have relevant expertise	6 (15)
	Advocacy, nonprofit: any organization that advocates and/or provides services on behalf of the public but does not fall into any other category (eg, American Cancer Society)	5 (13)
	Cancer center: any organization whose primary purpose is cancer prevention and treatment (eg, Fox Chase Cancer Center)	2 (5)
	General health information website: websites that do not provide services but provide health information (eg, WebMD, Yahoo! Health)	1 (3)
	Other, unspecified: websites that do not fall under any existing criteria	3 (8)

^a^In most instances, the text in this column represents the explanation from the coding scheme.

^b^Categories are not mutually exclusive.

^c^The 5 *other* cancer types were blood, gastroesophageal, oral, renal, and bladder cancer.

^d^In all but one case, the organization who hosted the website also developed the website.

### Accessibility and Credibility

As shown in Table 3, undefined medical terminology was widespread: close to 90% (35/39) of websites included technical language that a layperson may find difficult to understand without additional searching for information or help from a medical professional (eg, *biopsy* or *mastectomy*). However, only 23% (9/39) of websites had a statement indicating that medical professionals were the intended audience. Over half (23/39, 59%) of the websites indicated the scientific model used to calculate users’ cancer risk and most provided contact information for users to ask follow-up questions about either their risk or the website (32/39, 82%). Most websites simply provided information (31/39, 80%), but a subset also appeared to attempt to recruit patients (5/39, 13% either allowed or required individuals to enter their names and contact information if they wished to be contacted by a provider) or sell products (3/39, 8% provided links to purchase materials). Most websites referred users to a doctor (34/39, 87%) and provided links to additional websites with cancer-related information (32/39, 82%).

**Table 3:** Accessibility and credibility of internet-based cancer risk assessment tools (N=39 websites).

Website characteristics and description or examples of code^a^		Value, n (%)
**Limited accessibility for the general public: the information was not provided in a way that can be easily understood by the general public**		
	Undefined medical terminology: medical terminology that was not defined was included anywhere on the website (eg, ductal carcinoma in situ and biopsy)	35 (90)
	Professionals only: explicit statement that the website was intended for use by medical professionals	9 (23)
	Non-English option: a version of the risk assessment tool in a language other than English was provided (eg, Spanish-language version)	4 (10)
**Website transparency^b^: information that allows the user to obtain information about the quality of the website**		
	Contact information^c^: provides information about how to contact the developers of the website	32 (82)
	Indication of scientific basis of model: provides citation to peer-reviewed article about the risk prediction model; indicates which model was used (eg, Gail Model for breast cancer)	23 (59)
	Last modified^c^: indicates when website was last modified	11 (28)
**Underlying purpose^c^: the apparent goal of the website**		
	Information provision: provides risk information to website users without trying to recruit them as patients or advertise products	31 (80)
	Recruit website users: provides risk information with the apparent goal of recruiting website users to use their services (eg, requires user to provide contact information before receiving risk results and asking people to fill out form with contact information if they needed a provider)	5 (13)
	Sell products: provides risk information with the apparent goal of convincing users to purchase a product of theirs (eg, provide a link to purchase a book about cancer)	3 (8)
**Additional resources^b,c^: details about where to seek more information**		
	Doctor: website advises user to seek professional assistance (eg, talk to your doctor about your risk information)	34 (87)
	Links to additional websites: website provides links to locations to obtain additional cancer information (eg, to the American Cancer Society or National Cancer Institute)	32 (82)
	Social media: website had a link to one or more social media pages (eg, Facebook and Twitter)	18 (46)

^a^In most instances, the text in this column represents the explanation from the coding scheme.

^b^Categories are not mutually exclusive.

^c^Code not present in the study by Waters et al [9].

### Risk Communication Formats and Strategies

There was a wide variation in adherence to risk communication formats and strategies recommended for both informed decision making and encouraging behavior change. As shown in Table 4, a majority of websites followed best practice recommendations to specify a duration of risk (22/39, 56%), to provide absolute risk estimates (31/39, 80%), and to provide information about risk reduction strategies (27/39, 69%). Of the websites that provided quantitative risk information (23/39, 59%), all of them used percentages and/or natural frequencies, as recommended, and none of them used formats that are difficult for the general public to understand (eg, 1 in N, number needed to treat, and odds ratio). Very few websites followed recommendations to add qualitative labels to quantitative information (6/39, 15% of all websites and 6/23, 26% of websites that provided numbers), and only a small minority of websites framed risk information in both positive and negative terms (2/39, 5%). Consistent with recommendations, the single website that provided relative risk information also provided absolute risk information. The websites that included a visual display (20/39, 51%)—a strategy that is recommended to increase comprehension—tended to use recommended displays such as bar graphs (6/39, 15%) or icon arrays (also called stick figures; 4/39, 10%), but some websites used more difficult displays such as pie charts (3/39, 8%), line graphs (2/39, 5%), or speedometers or other less common displays (4/39, 10%). A solid majority of websites presented statements acknowledging uncertainty (23/39, 59%), as recommended. Most often, these statements indicated that the risk estimate is uncertain (19/39, 49%) or acknowledged the difficulty of estimating an individual’s risk using population-based data (15/39, 39%). Few websites provided a range for the estimate or confidence intervals (2/39, 5%).

Formats and strategies that can be useful for encouraging behavior change were present in less than half of the websites: 44% (17/39) provided social comparison information and 8% (3/39) compared the risk of cancer with other hazards. Several websites (16/39, 41%) also adopted the controversial practice of providing qualitative information but not quantitative information [48] (Table 4).

**Table 4:** Frequency of risk communication formats and strategies used by internet-based cancer risk assessment tools (N=39 websites).

Formats and strategies		Description and/or examples of code^a^	Value, n (%)
Duration of risk		Risk information specifies the timeframe for which it applies (eg, your risk of developing melanoma in the next 5 years has been estimated as very high)	22 (56)
Absolute risk		Risk estimate includes the baseline likelihood that the individual will develop a disease; can be presented with either quantitative or numeric risk estimates or qualitative or verbal labels	31 (80)
**Quantitative risk^b^**		Risk estimate included numbers	23 (59)
	Percent (%)	N/A^c^	23 (59)
	N in 100	N/A	5 (13)
	1 in N	N/A	0
	Number needed to treat	N/A	0
	Risk score	N/A	0
	Relative risk	N/A	1 (3)
	Odds	N/A	0
	None provided	No quantitative risk estimate was presented	16 (41)
Qualitative risk		Risk estimate included a verbal description of risk (eg, very large)	22 (56)
Both quantitative and qualitative risk		Risk information was presented using both numbers and verbal description	6 (15)
Positive framing		Risk estimate presented as one’s risk of not getting cancer (eg, this means you have a 99.2% chance of not getting cancer)	2 (5)
**Visual display^b^**		Whether or not there is a visual depiction of risk	20 (51)
	Color^d^	Risk information is represented by at least one color	20 (51)
	Bar graph^d^	Risk information presented using a bar graph	6 (15)
	Icons or stick figures^d^	Risk information presented using icons (eg, smiley faces) or stick figures	4 (10)
	Pie chart^d^	Risk information presented using a pie chart	3 (8)
	Line graph^d^	Risk information presented using a line graph	2 (5)
	Risk ladder^d^	Risk information presented in comparison with other risks on a vertical continuum	0
	Table^d^	Risk information presented using a table of numbers	0
	Other^d^	Risk information presented in a visual way not described above (eg, a needle on a dial pointing to where a user’s risk falls)	4 (10)
**Acknowledges uncertainty^b^**		Website indicates there may be error or uncertainty in the risk estimate	23 (59)
	Emphasizes estimate	Mentions that there is uncertainty about the accuracy of the risk estimate (eg, this is only an estimate, your actual risk may be higher or lower; the model or estimate is incomplete)	19 (49)
	Individualizing population risk	Mentions the difficulty of drawing conclusions about an individual from population-based data (eg, risk is based on population, not individual; cannot determine which individuals will or will not get cancer)	15 (39)
	Range or confidence intervals^d^	Provides a range of possible estimates (eg, 1.0%-2.0%)	2 (5)
Risk reduction strategies		Website provides information about how a user can reduce their risk of getting cancer (eg, quitting smoking)	27 (69)
**Social comparison^b^**		The website compares the user’s risk with the risk of other people	17 (44)
	Direct comparative^d^	The estimate is provided in a way that explicitly mentions how the user’s risk compares with others (eg, your risk is higher than average)	13 (33)
	Indirect comparative^d^	Separate information about the user’s risk and the average person’s risk is required in such a way that the user must make the comparison to determine how their risk compares with others (eg, your risk is 2%; the average woman’s risk is 5%)	8 (21)
Compared with other hazards		The risk of cancer is compared with the risk of other hazards (eg, your risk of breast cancer is 10%. The risk of being involved in a car accident is 20%)	3 (8)

^a^In most instances, the text in this column represents the explanation from the coding scheme.

^b^Categories are not mutually exclusive; individual websites may have received more than one code in this category.

^c^N/A: not applicable.

^d^Code not present in the study by Waters et al [9].

## Discussion

### Principal Findings

Despite a decade’s worth of risk communication research being conducted between our 2009 report of internet-based risk assessment tools and the investigation presented here, we found that many websites that host risk assessment tools continue to communicate information in such a way that is likely to be confusing or misunderstood. Therefore, this limits the effective translation of risk prediction models into risk assessment tools [46]. There are 3 key limitations.

First, several communication strategies limit accessibility to the general public. Although few websites state that they are only intended for medical professionals, the vast majority include medical terminology without explaining what it means. This finding is consistent with other research indicating that most internet-based risk assessment tools are difficult to use for individuals with limited health literacy [54].

Second, consistent with previous work by ourselves and others [9,18], the websites that develop and host the risk assessment tools typically do not provide sufficient information for people to determine the credibility of the tools. Although all but one website we reviewed indicated who developed it, and most websites provided a way to contact the developers in case of questions, not all websites provided information about the risk prediction model that was used to calculate their risk or provided information about when the website was last updated. Thus, users may find it difficult to determine the extent to which their personalized estimate is scientifically valid and/or personally relevant [18] and may instead rely on the credibility of the website as a heuristic to determine the credibility of the tool [55].

Third, although the use of some risk communication formats and strategies known to foster unbiased comprehension were more common than they were in 2009 (eg, using either percentages or natural frequency format to communicate quantitative risk estimates), others remain uncommon (eg, including qualitative labels when communicating quantitative risk estimates). This observation suggests that lessons learned about risk communication formats and strategies that are published in widely read journals [20,23,51] are not reaching organizations that develop and/or host websites that include risk assessment tools. This is concerning because as more risk prediction models are being developed, there is an increased likelihood of them being translated into a risk assessment tool and posted on the internet in the absence of guidance from risk communication experts [46].

One notable finding was that a considerable minority of websites used a controversial risk communication strategy that involved providing qualitative risk category labels but not quantitative risk estimates. This strategy is seen by some researchers as one that should not be used because the variability in people’s interpretations of qualitative labels impedes unbiased interpretations of risk information and informed decision making [25]. However, other researchers assert that, in circumstances wherein risk information is intended to promote behavior change, only qualitative categorical information is needed, not quantitative risk estimates [48]. This latter perspective is generally consistent with health communication and promotion campaigns that inform people that engaging in a particular behavior will increase their risk, but they do not provide details about the exact extent to which their risk is increased [56]. Providing qualitative labels in the absence of quantitative data may also overcome people’s tendencies to view any percentage estimate of less than 20% as a small risk [29]. This may be especially useful in situations in which researchers attempt to promote lifestyle behaviors to reduce cancer risk among healthy populations, who seldom have lifetime cancer risks of more than 20% in the absence of a high-penetrance genetic variant.

We also report 2 important differences between the work reported here and the work reported in 2009. First, 41 of the 47 websites examined in the 2009 review were no longer accessible or usable at their original URLs. Indeed, one of the websites we identified for this review was no longer accessible after 6 months. Second, this review identified several websites and tools that appeared to be developed and/or hosted by individuals who indicated they had medical or scientific expertise, but most did not appear to be affiliated with a health care institution or university. Both these findings point to the potential difficulty of verifying the validity of the risk prediction models over time. In addition, independent experts without formal affiliations to scientific institutions may have less access to collaborators with expertise in risk prediction modeling, risk communication, and behavior change than developers affiliated with governmental, educational, or health care institutions.

### Limitations and Future Directions

It is possible that the initial search overlooked some relevant websites or the triage process miscategorized sites. However, our process ensured that if there were any doubts about the relevance of a website or tool, at least one other member of the coding team was consulted. We did not involve patients or members of the public in the assessment of the websites. Future research should consider doing so to increase the applicability of the results to these key end users. We also engaged in double coding for the websites included in this report; a third coder acted as a tiebreaker in particularly difficult circumstances. Unfortunately, we do not have data about how often each risk assessment tool was accessed, who accesses them, or what users do with the information they obtain from the tools. Future research should investigate this issue. We considered creating a summary score indicating how many risk communication formats and strategies were used per site, but determined that doing so was unwise because there is little research examining how many formats or strategies should be used and in which combinations. Several researchers have determined that simpler communications are either more easily understood or just as easily understood as more complex communications [37,57,58], but future research should determine which formats and strategies should be prioritized. Finally, the number of websites was too small to determine whether there were statistically significant differences between the website affiliation category and the use of risk communication formats or strategies.

### Conclusions

This study found extensive variability in the extent to which internet-based risk assessment tools and the websites that host them provide information in a way that facilitates understanding among the general population. Gaps in information accessibility, in the availability of information needed to evaluate the website’s credibility, and in the use of risk communication formats and strategies that foster comprehension limit the translation of risk prediction models to wider public health practice [46]. Improving the quality of communication of such tools will likely limit confusion and foster an understanding of the information among users.

## Appendix

Multimedia Appendix 1 Websites hosting internet-based cancer risk assessment tools.

## Abbreviations

HINTS: Health Information National Trends Survey
NCI: National Cancer Institute
