# Supplementary material for: Adherence of Internet-Based Cancer Risk Assessment Tools to Best Practices in Risk Communication: Content Analysis
Source: J Med Internet Res. 2021 Jan 25;23(1):e23318. (PMC7870349)
Supplement: Multimedia Appendix 1 [file jmir_v23i1e23318_app1.docx]

Multimedia Appendix 1. Websites hosting Internet-based cancer risk assessment tools.

| 1 | AlfredHealth  <https://www.alfredhealth.org.au/melanoma-risk-calculator/public>  <https://perma.cc/XS3R-UN3C> |
| --- | --- |
| 2 | Amazon Web Services ^a^  <http://lungcancerrisk.s3-website-us-east-1.amazonaws.com/>  <https://perma.cc/2YGY-7989> |
| 3 | American Association for Thoracic Surgery  <https://www.aats.org/aatsimis/AATSWeb/Resources/Lung_Cancer_Screening/AATSWeb/Association/About/Resources/Lung_Cancer_Risk_Assessment_Tool.aspx?hkey=29f118a8-d7a6-4bcd-a9b3-7e85484881b8>  <https://perma.cc/2BNS-LJFP> |
| 4 | Bowel Cancer New Zealand ^b^  <https://bowelcancernz.org.nz/about-bowel-cancer/risk-calculator/> |
| 5 | Breast Cancer Surveillance Consortium  <https://tools.bcsc-scc.org/bc5yearrisk/calculator.htm>  <https://perma.cc/L8XV-K85C> |
| 6 | Bright Pink ^d^  <https://www.assessyourrisk.org/>  <https://perma.cc/UXN7-UPC5> |
| 7 | Cancer Care Ontario ^e^  <https://www.mycanceriq.ca/Cancers/Risk>  <https://perma.cc/N9R6-3BGH> |
| 8 | CareFirst ^c^  <http://carefirst.staywellsolutionsonline.com/InteractiveTools/RiskAssessments/42,BreastCancerRisk>  <https://perma.cc/HP7W-5VMY> |
| 9 | CareFirst  <http://carefirst.staywellsolutionsonline.com/InteractiveTools/RiskAssessments/42,ColorectalCancerRisk>  <https://perma.cc/4J6C-6VX3> |
| 10 | Cedars Sinai  <https://www.cedars-sinai.org/health-library/risk-assessments/colorectal-cancer-risk-assessment.html>  <https://perma.cc/E4JQ-FKXA> |
| 11 | Cleveland Clinic  <https://digestive.ccf.org/>  <https://perma.cc/4ND9-HXUD> |
| 12 | ClinRisk (10 year) ^d^  <https://www.qcancer.org/>  <https://perma.cc/X2BP-A8EK> |
| 13 | ClinRisk (15 year)  <https://www.qcancer.org/>  <https://perma.cc/N2CC-RJVA> |
| 14 | Estronaut ^c^  <http://www.estronaut.com/a/breastInteractive2.htm>  <https://perma.cc/J3CF-YEYY> |
| 15 | Health24  <https://www.health24.com/Medical/Cancer/Tools/Cancer-are-you-at-risk-20130203>  <https://perma.cc/J549-94WU> |
| 16 | Henry Ford Health System  <https://www.henryford.com/health-risk-assessments>  <https://perma.cc/7ZYU-FVDN> |
| 17 | MDCalc  <https://www.mdcalc.com/gail-model-breast-cancer-risk>  <https://perma.cc/TD6G-9QDL> |
| 18 | MensXMachina Research Lab  <http://mensxmachina.org/en/hunt-ntnu-lung-cancer-risk-calculator/>  <https://perma.cc/2DX6-LLMY> |
| 19 | MetaOptima  <https://molescope.com/risk-assessment/>  <https://perma.cc/EQC3-NFQR> |
| 20 | Norton Healthcare  <https://nortonhealthcare.com/services-and-conditions/prevention-and-wellness/health-risk-assessments>  <https://perma.cc/2L6Y-4CRS> |
| 21 | Norwest Skin Cancer Centre  <http://www.skincancerclinics.net.au/risk/skin-cancer-risk-calculator>  <https://perma.cc/7284-AJQQ> |
| 22 | Omni Calculator  <https://www.omnicalculator.com/health/lung-cancer-risk-calculator-for-smokers>  <https://perma.cc/L3ED-4G52> |
| 23 | Oncimmune  <https://oncimmune.com/calculate-lung-cancer-risk/>  <https://perma.cc/9MBX-KWA7> |
| 24 | Pancreatic Cancer Action Network  <https://www.pancan.org/facing-pancreatic-cancer/about-pancreatic-cancer/risk-factors/risk-assessment-test/>  <https://perma.cc/J8BY-LG59> |
| 25 | Peter MacCallum Cancer Center  <https://www.petermac.org/iprevent>  <https://perma.cc/6Q7E-ZR5Z> |
| 26 | Robert Nam, MD FRCSC  <https://www.prostaterisk.ca/>  <https://perma.cc/KMH2-AYUJ> |
| 27 | Siteman Cancer Center ^c, e^  <https://siteman.wustl.edu/prevention/ydr/>  <https://perma.cc/VF3M-77DE> |
| 28 | Steven B. Halls, MD ^c^  <https://halls.md/breast/risk.htm>  <https://perma.cc/US3F-BFNH> |
| 29 | The Prostate Cancer Research Foundation  <http://www.prostatecancer-riskcalculator.com/>  <https://perma.cc/3S4N-JGRN> |
| 30 | The Truth About Cancer  <https://cancerquiz.org/>  <https://perma.cc/6UYC-7QEH> |
| 31 | UCLA Health  <http://healthinfo.uclahealth.org/42,ColorectalCancerRisk>  <https://perma.cc/72Z2-R3SU> |
| 32 | University of Michigan  <https://shouldiscreen.com/English/lung-cancer-risk-calculator>  <https://perma.cc/P2HF-ZE96> |
| 33 | University of Rochester Medical Center  <https://www.urmc.rochester.edu/encyclopedia/content.aspx?contenttypeid=42&contentid=ColorectalCancerRisk>  <https://perma.cc/86NR-VMC3> |
| 34 | US National Cancer Institute ^c^  <https://bcrisktool.cancer.gov/>  <https://perma.cc/T5EX-2X4L> |
| 35 | US National Cancer Institute ^b^  <https://colo.cancer.gov/colorectalcancerrisk/> |
| 36 | US National Cancer Institute  <https://ccrisktool.cancer.gov/index.html>  <https://perma.cc/QF8T-A2PU> |
| 37 | US National Cancer Institute  <https://analysistools.nci.nih.gov/lungCancerRiskAssessment/#/>  <https://perma.cc/GTA5-26PX> |
| 38 | US National Cancer Institute ^c^  <https://mrisktool.cancer.gov/>  <https://perma.cc/PUX8-7Y9B> |
| 39 | Wolfson Institute of Preventive Medicine, Queen Mary University of London  <https://ibis.ikonopedia.com/>  <https://perma.cc/DNL4-4WYH> |

^a^ Denotes instances in which the sponsor of the website is unclear.

^b^ Denotes instances in which the website was taken down after initial coding, but prior to the creation of a permalink.

^c^ Indicates website present in both 2009 review and current review.

^d^ Denotes instances in which the website hosted a single calculator that reported a user’s risk of more than one cancer type.

^e^ Denotes instances in which the website hosted more than one calculator (allowing the user to choose what type of cancer they calculated their risk for).
